# Phytopathogen type III effectors as probes of biological systems

**DOI:** 10.1111/1751-7915.12042

**Published:** 2013-02-25

**Authors:** Amy Huei-Yi Lee, Maggie A Middleton, David S Guttman, Darrell Desveaux

**Affiliations:** 1 Department of Cell & Systems Biology, University of TorontoToronto, Ontario, Canada; 2 Centre for the Analysis of Genome Evolution & Function, University of TorontoToronto, Ontario, Canada

## Abstract

Bacterial phytopathogens utilize a myriad of virulence factors to modulate their plant hosts in order to promote successful pathogenesis. One potent virulence strategy is to inject these virulence proteins into plant cells via the type III secretion system. Characterizing the host targets and the molecular mechanisms of type III secreted proteins, known as effectors, has illuminated our understanding of eukaryotic cell biology. As a result, these effectors can serve as molecular probes to aid in our understanding of plant cellular processes, such as immune signalling, vesicle trafficking, cytoskeleton stability and transcriptional regulation. Furthermore, given that effectors directly and specifically interact with their targets within plant cells, these virulence proteins have enormous biotechnological potential for manipulating eukaryotic systems.

An important strategy employed by successful bacterial pathogens is to specifically attack key intracellular host processes to allow for the maximal proliferation of the pathogen. Many Gram-negative bacterial phytopathogens achieve this goal by delivering virulence proteins, or effectors, into the host cytosol using the type III secretion system (Buttner and Bonas, 2003; Jin *et al*., 2003; Cornelis, 2006; Zhou and Chai, 2008; Lewis *et al*., 2009; Deslandes and Rivas, 2012). Once inside plant cells, type III secreted effectors (T3SEs) can subvert signalling pathways, modulate transcription, hijack intracellular transport, modify the cytoskeleton and suppress host defences (Bhavsar *et al*., 2007; Hann *et al*., 2010; Cui and Shao, 2011; Lewis *et al*., 2011; Deslandes and Rivas, 2012; Lindeberg *et al*., 2012). Elucidating the mechanisms by which these T3SEs promote bacterial virulence has furthered our understanding of eukaryotic cell biology. Specifically, studying these effector proteins has allowed us to (i) uncover novel components of specific cellular processes by identifying effector targets; (ii) characterize the molecular functions of these novel components; and (iii) utilize these effectors as biotechnology tools to manipulate eukaryotic cellular processes. In the following sections, we will highlight a number of bacterial phytopathogen T3SEs with known host targets and how characterizing effector function has provided insight into conserved cellular processes such as kinase signalling, intracellular trafficking, cytoskeleton and transcriptional regulation in plants (Fig. 1). In addition, we will discuss a number of biotechnological applications utilizing effector proteins.

**Figure 1:**
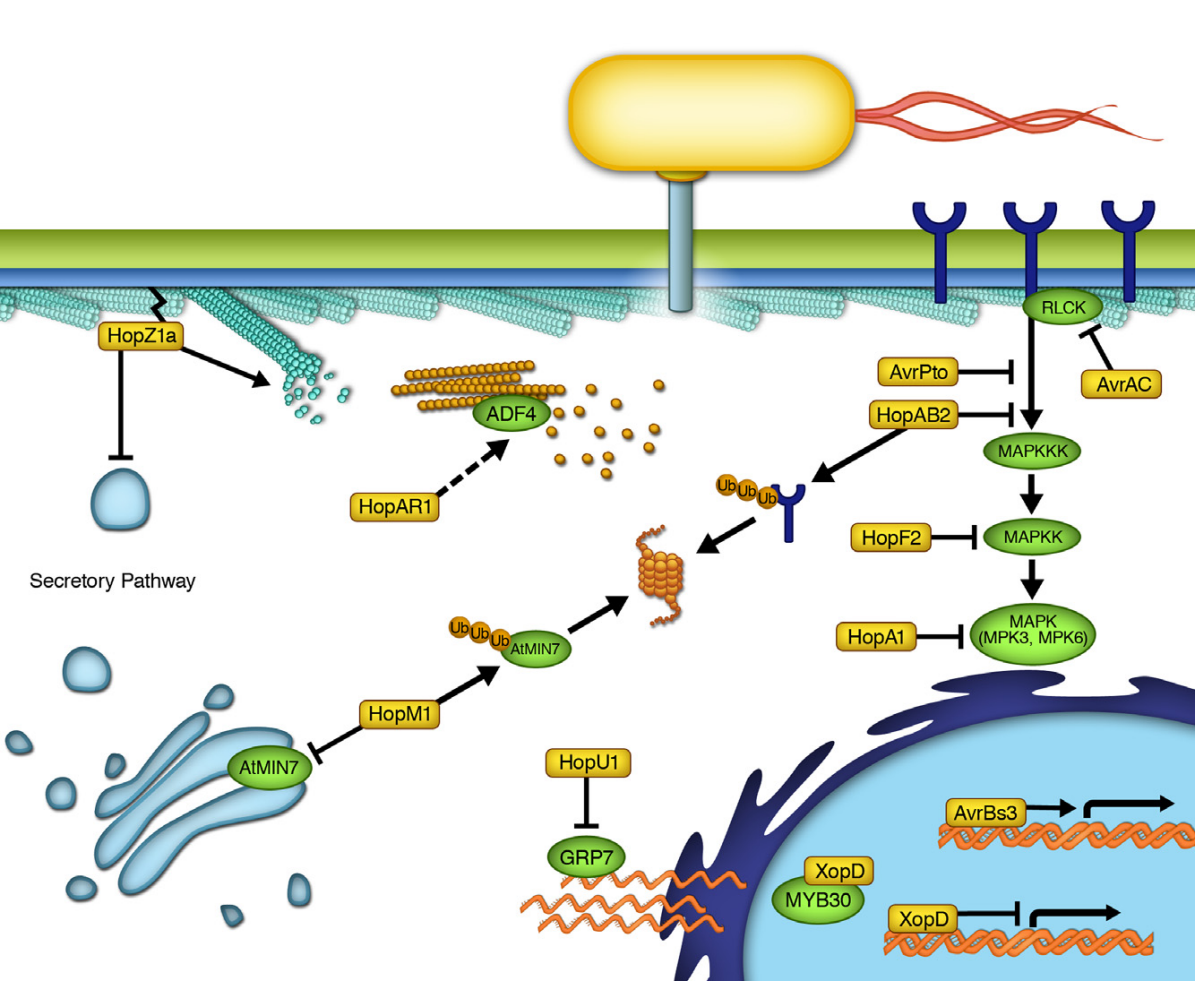
Eukaryotic cellular systems targeted by phytopathogenic type III secreted effectors (T3SEs). Bacterial phytopathogens use T3SEs (yellow boxes) to inhibit upstream signalling components such as targeting FLS2 for proteasome degradation (AvrPtoB), prevent the phosphorylation of BAK1 (AvrAC and AvrPto), or degrade BIK1 (HopAR1). In addition, phytopathogen T3SEs can inhibit the phosphorylation of downstream signalling components such as the MAPKKs (HopF2) and MAPKs (HopAI1). Two nuclear-localized T3SEs, AvrBs3 and XopD, bind to DNA and alter transcription in plant cells. Specifically, AvrBS3 is a transcription activator that binds to the UPA box of its target genes. XopD represses the activities of a eukaryotic transcription factor, MYB30, which consequently suppresses the transcription of plant immune response genes. HopU1 targets RNA-binding proteins such as AtGRP7, which alters RNA processing in the plant host. HopM1 targets a plant ARF–GEF (AtMIN7) for degradation by the proteasome and consequently inhibit the secretory pathway. HopZ1a is the first bacterial phytopathogen T3SE shown to bind to plant tubulin and causes microtubule destruction. In addition, HopZ1a inhibits the plant secretory pathway. Lastly, the HopAR1-elicited plant immune response requires an actin regulator, ADF4. However, the link between HopAR1 and ADF4 or HopAR1 and actin is currently unclear.

## Bacterial type III effectors that target MAPK signalling

**Figure 2:**
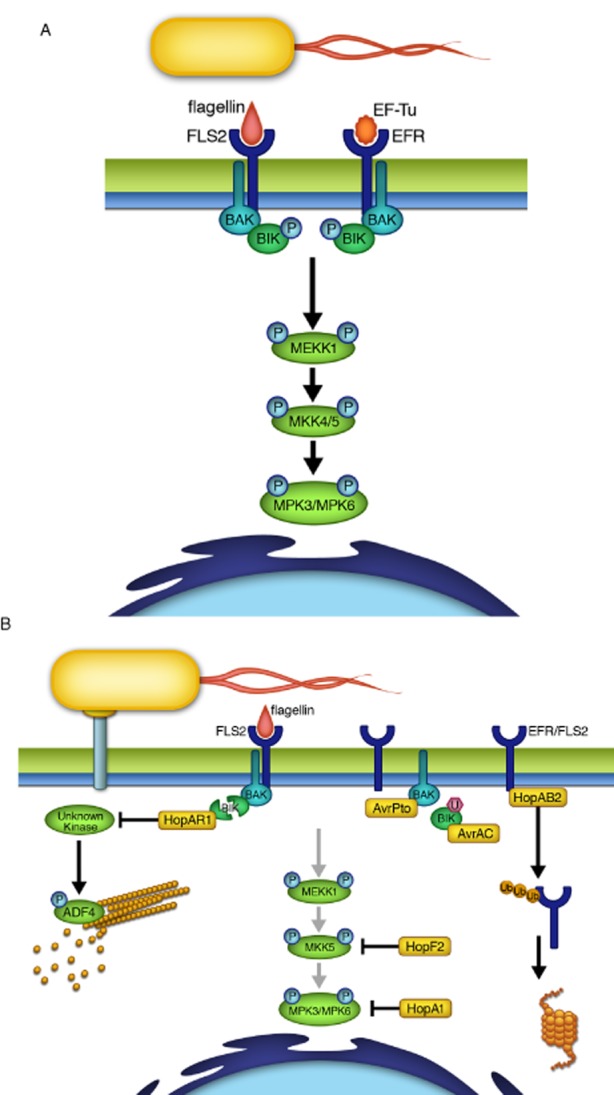
Phytopathogen T3SEs disrupt host-signalling pathways to suppress immune responses. A. Pattern recognition receptors such as FLS2 and EFR allow the plant host to recognize microbe-associated molecular patterns (MAMPs), such as bacterial flagellin and elongation factor Tu (EF-Tu) respectively. This MAMP recognition leads to the activation of signalling cascades via a series of phosphorylation events, which subsequently activates plant immunity. B. Bacterial phytopathogens use T3SEs (yellow boxes) to inhibit upstream signalling components. This includes the targeting of FLS2 for proteasome degradation (HopAB2), preventing the phosphorylation of BAK1 (AvrAC and AvrPto), or directly degrading BIK1 (HopAR1). In addition, phytopathogen T3SEs can inhibit the phosphorylation of downstream signalling components such as the MAPKKs (HopF2) and MAPKs (HopAI1).

In order to mount an effective immune response against microbes, plant cells utilize pattern-recognition receptors (PRRs) to recognize various microbe-associated molecular patterns (MAMPs) (Schwessinger and Zipfel, 2008; Zipfel, 2008; Zhang and Zhou, 2010; Segonzac and Zipfel, 2011; Beck *et al*., 2012; Schwessinger and Ronald, 2012). Upon the perception of MAMPs such as bacterial flagellin or EF-Tu, PRRs form a heterodimer with a regulatory leucine-rich repeat receptor-like kinase (LRR-RLK), known as BAK1 (Chinchilla *et al*., 2007; Heese *et al*., 2007; Roux *et al*., 2011). Subsequently, this PRR-BAK1 heterodimer formation leads to phosphorylation of both the PRR and BAK1 (Chinchilla *et al*., 2007; Heese *et al*., 2007; Schulze *et al*., 2010; Roux *et al*., 2011). BAK1 then transmits the signal by phosphorylating a receptor-like cytoplasmic kinase (RLCK), BIK1 (Lu *et al*., 2010). In the case of flagellin perception by the PRR, FLS2, activated BIK1 transphosphorylates BAK1 and FLS2, presumably as a way to amplify the MAMP signal (Lu *et al*., 2010). Additionally, activated BIK1 is likely released from the FLS2–BAK1 complex in order to activate downstream signalling components (Fig. 2A) (Lu *et al*., 2010; Zhang *et al*., 2007; Zhang and Zhou, 2010).

One of these downstream signalling components is the mitogen-activated protein kinase (MAPK) signalling cascade (Fig. 2A). The organization of plant MAPK pathways follows the canonical architecture, where the three types of kinases, MAPKKK, MAPKK and MAPK, are sequentially activated by phosphorylation. There are two distinct MAPK pathways that regulate plant immunity: the MAPK4 pathway, which negatively regulates defences, and the MAPK3/6 pathway that positively regulates plant immunity (Asai *et al*., 2002; Nicaise *et al*., 2009; Pitzschke *et al*., 2009; Bernoux *et al*., 2011; Tena *et al*., 2011).

Characterization of two unrelated T3SEs from the phytopathogenic bacterium *Pseudomonas syringae*, AvrPto and HopAB2 (previously known as AvrPtoB), has refined our understanding of plant immune signalling (Fig. 2B). Both AvrPto and HopAB2 prevent immune signalling by targeting upstream components FLS2, EFR and the co-receptor BAK1 (Gohre *et al*., 2008; Xiang *et al*., 2008); however, the mechanisms by which these two effectors target the PRRs are different. HopAB2 is a bacterial-encoded E3 ubiquitin ligase that targets FLS2 and EFR for degradation by the host ubiquitination pathway (Rosebrock *et al*., 2007; Gohre *et al*., 2008). On the other hand, AvrPto is proposed to target the co-receptor BAK1 rather than the individual PRRs (Shan *et al*., 2008). Specifically, Shan and colleagues show that AvrPto inhibits FLS2 activation by binding to BAK1 and disrupting the FLS2–BAK1 heterodimer formation (Shan *et al*., 2008). The FLS2–BAK1 interaction upon MAMP perception is a critical step of MAMP-triggered immunity (MTI) and leads to the phosphorylation of both FLS2 and BAK1 (Chinchilla *et al*., 2007; Heese *et al*., 2007; Schulze *et al*., 2010; Roux *et al*., 2011). Thus, by preventing the formation of FLS2–BAK1 complex, AvrPto inhibits the MTI activation demonstrating that the interaction between FLS2 and BAK1 is critical for MTI signalling.

Another way for bacterial phytopathogens to block immune signalling is to target the RLCKs; consequently, bacterial T3SEs can be used to identify novel RLCKs Involved in plant immunity. *P. syringae* has been shown to target *Arabidopsis* RLCKs via the cysteine protease T3SE HopAR1 (previously known as AvrPphB) (Shao *et al*., 2003; Ade *et al*., 2007; Zhang *et al*., 2007). Specifically, HopAR1 inhibits plant immunity by proteolytically cleaving various RLCKs, including PBS1 and PBS1-like (PBLs) proteins such as BIK1 (Fig. 2B) (Shao *et al*., 2003; Ade *et al*., 2007; Zhang *et al*., 2007). On the other hand, *Xanthomonas campestris* pv. *campestris* (Xcc) utilizes the T3SE AvrAC to interact with two related *Arabidopsis* immune signalling RLCKs, BIK1 and RIPK, to inhibit their phosphorylation (Fig. 2B) (Feng *et al*., 2012). AvrAC covalently modifies BIK1 and RIPK via the addition of uridine 5′-monophosphate (UMP) on the serine and threonine residues of the kinase activation loops (Feng *et al*., 2012), and thereby promotes bacterial virulence by inhibiting the phosphorylation of BIK1 and RIPK. Together, these effectors have demonstrated that the protein stability and the phosphorylation state of RLCKs are important for subsequent immune signalling.

*Pseudomonas syringae* also uses T3SEs to directly target host MAPK pathways (Fig. 2B). Characterization of the molecular mechanisms by which two T3SEs, HopAI1 and HopF2, modify their MAPK targets in plants has led to the identification of key functional residues in MAPK signalling components. For instance, HopAI1 directly interacts with MPK3 and MPK6 *in planta* (Zhang *et al*., 2007). HopAI1 is a bacterial-encoded phosphothreonine lyase that irreversibly dephosphorylates its plant MAPK targets, MPK3 and MPK6, at the canonical phosphorylation sites such that they cannot be re-phosphorylated (Li *et al*., 2007; Zhang *et al*., 2007). Another *P. syringae* effector that targets the plant MAPK pathway is HopF2 (Wang *et al*., 2010). HopF2 interacts with a number of *Arabidopsis* MAPKKs, including MKK3/4/5/6/10 (Wang *et al*., 2010). Previous work has shown that the HopF homologue HopF1 has structural similarity to the ADP-ribosyltransferase (ADP-RT) domain of diphtheria toxin (Singer *et al*., 2004). HopF2 requires the conserved ADP-RT catalytic residues (R71 and D175) to both bind to and modify the target, MKK5 (Wang *et al*., 2010). By identifying the ADP-RT site of HopF2 on MKK5, Wang and colleagues uncovered a conserved arginine residue (R313) that is important for MKK5 function (Wang *et al*., 2010).

Given that effectors can irreversibly modify their specific kinase targets as described above, these effectors can be used as kinase inhibitors to study eukaryotic signal transduction. For example, T3SEs from animal pathogens that also target the MAPK pathways, such as *Shigella* OspF and *Yersinia* YopH, have been used to generate synthetic pathways and study pathway behaviour in response to stimuli (Wei *et al*., 2012). OspF is a phosphothreonine lyase that blocks MAPK signalling in both yeast and mammalian cells by irreversibly inactivating the MAPK targets. By generating an OspF mutant (ΔN-OspF) lacking its canonical docking peptide, Wei and colleagues (2012) could target OspF to specific MAPK pathway in yeast and characterize the alteration in pathway responses as a consequence of OspF inhibition. Thus, effectors that target signalling cascades can be utilized in synthetic biology to study cellular behaviour in response to rewired kinase pathways. Furthermore, given that OspF also targets MAPK signalling in human immune T-cells, OspF can be used as a synthetic ‘pause switch’ to transiently disable T-cell signalling (Wei *et al*., 2012). Therefore, bacterial effectors that target immune signalling pathways can be used to re-engineer and regulate signalling in immune cells, eventually leading to the development of better therapeutic options to treat cancer and chronic infection.

## Bacterial effectors that target intracellular trafficking

Plant cells deal with the presence of pathogens by delivering defence compounds such as antimicrobial proteins, phytoalexins and cell wall components to sites of infection via secretory pathways (Robatzek, 2007; Hoefle and Huckelhoven, 2008; Kwon *et al*., 2008; Bednarek *et al*., 2010; Wang and Dong, 2011; Beck *et al*., 2012; Yun and Kwon, 2012). The first *P. syringae* T3SE shown to interfere with plant secretion is the conserved effector, HopM1 (Fig. 1) (Nomura *et al*., 2006; 2011), which plays an important role in *P. syringae* virulence (Badel *et al*., 2003; DebRoy *et al*., 2004). The identification of HopM1 interactors in *Arabidopsis* uncovered a critical and novel secretory component that is required for cell wall-based defence responses. Using a truncated HopM1 as bait, Nomura and colleagues identified a number of eukaryotic interactors from an *Arabidopsis thaliana* cDNA library and named these interactors AtMINs (*A**rabidopsis* *t**haliana* HopM interactors) (Nomura *et al*., 2006). One of the HopM1 interactors, AtMIN7, is a previously uncharacterized ARF-guaninine nucleotide exchange factor (ARF–GEF) in *Arabidopsis* (Nomura *et al*., 2006; 2011). The interaction between HopM1 and AtMIN7 targets AtMIN7 for degradation by the plant proteasome (Nomura *et al*., 2006). Given that ARF–GEF proteins play an important role in regulating vesicle trafficking, Nomura and colleagues hypothesized that the virulence function of HopM1 is to inhibit the plant secretory pathway by destabilizing AtMIN7 (Nomura *et al*., 2006). In support of this, HopM1 and AtMIN7 co-localize to the trans-Golgi network/early endosome (Nomura *et al*., 2011). Using a secretion inhibitor that targets ARF–GEFs, brefeldin A (BFA), the authors demonstrate that BFA functionally mimics HopM1 and restores virulence to *hopM1* mutant bacteria. As a further support for HopM1 virulence function, the *hopM1* mutant cannot inhibit the secretory-dependent cell wall-based defences. Interestingly, HopM1 does not target all the ARF–GEF proteins in *Arabidopsis*, indicating that HopM1 may selectively target a subset of secretory pathways (Nomura *et al*., 2006; 2011).

Another *P. syringae* effector that was used to identify a probable secretion-associated protein was AvrPto (Speth *et al*., 2009). Using yeast two-hybrid screens, AvrPto has been shown to interact with GTP-bound small Rab GTPases (Bogdanove and Martin, 2000; Speth *et al*., 2009). RabE localizes to the Golgi and the plasma membrane of the plant cells (Speth *et al*., 2009); however, despite the co-localization of AvrPto and RabE to the plasma membrane, Speth and colleagues could not detect *in vivo* interaction between these two proteins (Speth *et al*., 2009). Nevertheless, RabE appears to play roles in plant immunity since there is a polarized accumulation of RabE during R-gene mediated defence (Speth *et al*., 2009). Additionally, *Arabidopsis* expressing RabE Q74L (the RabE mutant that is locked in the GTP-bound form) constitutively secretes PR1 proteins and is more resistant to virulent *P. syringae* (Speth *et al*., 2009). Thus, the RabE GTPase is likely involved in vesicle trafficking during the defence response.

## Bacterial effectors that target the cytoskeleton

The actin and microtubule cytoskeletons are essential for many cellular functions such as development, organelle movement and responses to biotic stresses (Schmidt and Panstruga, 2007; Day *et al*., 2011; Higaki *et al*., 2011; Smertenko and Franklin-Tong, 2011; Uma *et al*., 2011). Earlier work on fungal and oomycete pathogens have shown that the actin and microtubule cytoskeletons are remodelled in order to mount an appropriate defence response against these pathogens (Skalamera and Heath, 1998; Opalski *et al*., 2005; Schutz *et al*., 2006). Additionally, the proper recycling of the bacterial flagellin receptor, FLS2, is dependent on both the actin and the microtubule networks (Robatzek *et al*., 2006). Our understanding of the involvement of the plant cytoskeleton in mounting effective immune responses against bacterial pathogens has also benefited from the functional characterizing of two unrelated *P. syringae* T3SEs, HopAR1 and HopZ1a (Fig. 1).

Using reverse genetics, Tian and colleagues identified a novel actin-binding protein involved in the defence signalling triggered by the *P. syringae* effector HopAR1 (Tian *et al*., 2009). *Arabidopsis* plants expressing the resistance (R) protein, RPS5, recognize the activity of HopAR1 and consequently induces a strong immune response known as the effector-triggered immunity (ETI) (Ade *et al*., 2007). However, in plants lacking the actin-depolymerizing factor (ADF4), HopAR1 no longer induces ETI, presumably due to lower *RPS5* mRNA levels in the *adf4* mutant plants (Tian *et al*., 2009; Porter *et al*., 2012). Furthermore, *RPS5* transcript levels and the subsequent HopAR1-ETI induction are dependent on the phosphorylation of ADF4 (Porter *et al*., 2012). Additionally, the phosphorylation state of ADF4 alters its binding to the actin cytoskeleton (Porter *et al*., 2012). While the mechanism by which HopAR1 alters ADF4 phosphorylation is currently unknown, work on HopAR1 has nevertheless revealed a previously unknown link between the actin cytoskeleton plant immunity.

HopZ1a was recently identified as the first bacterial effector that interacts with plant tubulin (Lee *et al*., 2012). Importantly, this work has uncovered a novel role for the microtubule cytoskeleton in plant defence against bacterial pathogens. HopZ1a is an acetyltransferase that is activated by a eukaryotic cofactor, phytic acid. Activated HopZ1a acetylates both itself and tubulin *in vitro*, and causes destruction of *Arabidopsis* microtubule networks (Lee *et al*., 2012). The destruction of microtubules can promote bacterial virulence, as *P. syringae* grows significantly better in *Arabidopsis* plants treated with oryzalin, a microtubule disruptor (Lee *et al*., 2012). Interestingly, HopZ1a does not alter actin networks, suggesting that HopZ1a specifically targets microtubules. Although it is unclear if HopZ1a manipulates the plant microtubule networks directly or indirectly, HopZ1a does require its acetyltransferase activity to cause microtubule destruction, inhibit secretion and block cell wall-based defences in *Arabidopsis* (Lee *et al*., 2012). Future work characterizing the molecular mechanism by which HopZ1a modifies the microtubule networks will help clarify the link between microtubules, secretory pathways and immunity.

## Bacterial effectors that target transcription

**Figure 3:**
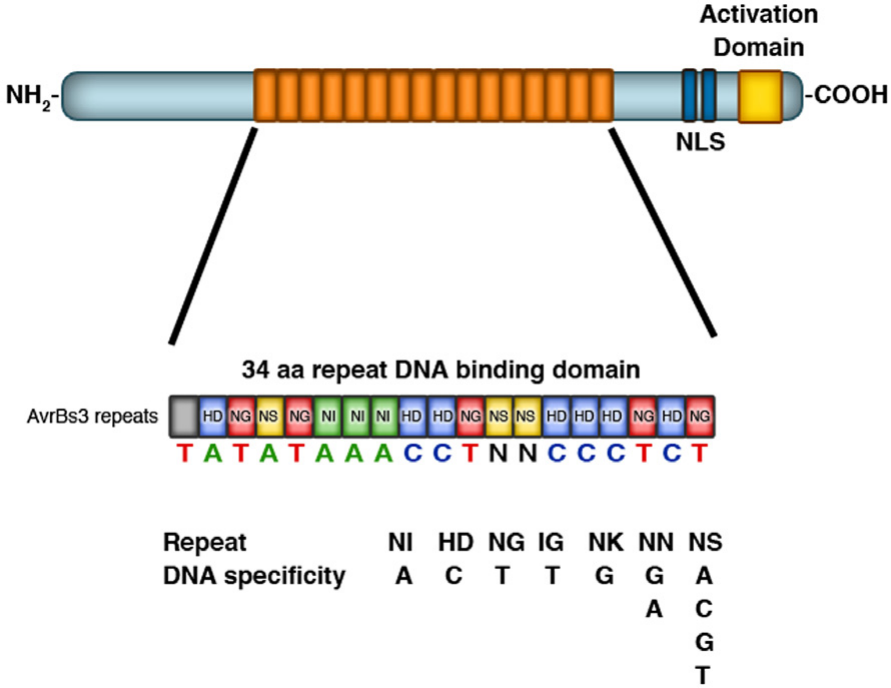
DNA binding specificity of TAL effectors. TAL effectors (TALEs) such as AvrBs3 are transcription activators that bind to the promoter of their target genes. TALEs contain a central domain with 17.5 repeats, a C-terminal nuclear localization signal (NLS) and the transcriptional activation domain. The DNA-binding specificity of TALEs is dependent on a 2-amino-acid motif within each repeat. As an example, the consensus *UPA* box and the corresponding 2-amino-acid motifs for AvrBs3 are illustrated, with the TALE DNA code shown below. Figure is adapted from Boch and Bonas (2010).

One of the best-characterized phytopathogen T3SEs shown to directly alter host transcription is the *Xanthomonas* T3SE AvrBs3 (Fig. 3) (Boch and Bonas, 2010). In susceptible host plants, AvrBs3 induces an enlargement of mesophyll cells, a phenomenon called hypertrophy (Marois *et al*., 2002). Earlier work demonstrated that AvrBs3 contains a functional C-terminal nuclear localization sequence (NLS) and a transcriptional activation domain (AD) that are both essential for AvrBS3 activity (Van den Ackerveken *et al*., 1996; Szurek *et al*., 2001; 2002; Gurlebeck *et al*., 2005). Additionally, AvrBs3 has a central domain that is highly repetitive, containing 17.5 nearly identical 34-amino-acid repeats (Marois *et al*., 2002). Given that AvrBs3 is localized to the nucleus and contains signatures of eukaryotic transcription factor, it has been suggested that AvrBs3 and its family members are transcription activator-like (TAL) proteins (Boch and Bonas, 2010). Indeed, AvrBs3 binds to the promoter of its target genes, named *upa* genes (upregulated by AvrBs3), and activates their transcription (Fig. 3) (Kay *et al*., 2007). These *upa* genes all share a conserved promoter element that AvrBs3 binds to, known as the UPA box (Kay *et al*., 2007). Importantly, the *in vitro* binding of AvrBs3 to one of its target genes, *upa*20, requires the central domain of AvrBs3 that contains the 17.5 repeats (Kay *et al*., 2007). The breakthrough to understanding how the repeat domain of AvrBs3 mediates DNA binding came from the observation that the number of AvrBs3-repeats roughly correspond to the nucleotide-length of the UPA box (Boch *et al*., 2009; Moscou and Bogdanove, 2009). The N-terminal repeats in AvrBs3 correspond to the 5′-end of the UPA box, while the C-terminal repeats of AvrBs3 correspond to the 3′-end of the UPA box (Boch *et al*., 2009; Moscou and Bogdanove, 2009). Careful analyses revealed that the specificity of each repeat is determined by 2-amino-acid motif, known as the repeat variable-di-residue (RVD), and the code for how TAL effectors (TALEs) bind to DNA was deciphered (Fig. 3) (Boch *et al*., 2009; Moscou and Bogdanove, 2009; Deng *et al*., 2012).

With the knowledge of how AvrBs3 and other TALEs bind to DNA, exciting new biotechnological applications have been developed and proposed. For instance, inserting different TALE-binding sites in the promoter of a particular R gene can generate transgenic plants that display broad resistance to *Xanthomonas* infection (Hummel *et al*., 2012). Given that many TALEs are critical to *Xanthomonas* virulence, one of the ways to combat *Xanthomonas* infection in the field is to identify plants with R genes against specific TALEs. However, traditional approaches of generating plants that constitutively express R proteins often lead to deleterious effects. In contrast, the addition of customized TALE-binding sites in the promoter of any R gene can generate resistant rice plants that are healthy and fertile, since the R proteins are only expressed in response to a particular TALE (Hummel *et al*., 2012).

Furthermore, the modular nature of the DNA-binding domain in TALEs provides a mechanism to manipulate genomes and modulate transcription in many eukaryotic systems (Sanjana *et al*., 2012). Importantly, the TALE code allows the generation of custom TALE DNA-binding domains with specificity, leading to endless possibilities for genome editing (Sanjana *et al*., 2012). TALE-TFs, which have the TALE DNA-binding domains coupled to transcription factors (TFs), modulate gene transcription at specific sites (Zhang *et al*., 2011; Mahfouz *et al*., 2012; Sanjana *et al*., 2012). On the other hand, TALENs, which result from the addition of endonucleases (ENs) to the TALE DNA binding-domains, promote site-specific insertion of any DNA of interest (Miller *et al*., 2011; Sanjana *et al*., 2012). Given the versatility and the specificity of designer TALENs, the journal *Nature Methods* selected TALENS, along with zinc-finger nucleases, as the ‘Method of Year 2011’ (Baker, 2012). Biotechnology companies such as Life Technologies and Cellectis now offer commercially available custom-made TALENs. Together, designer TALEs have provided new genomic toolbox that can be used in *Arabidopsis*, human, yeast, *Caenorhabditis elegans* and potentially any organism (Bogdanove and Voytas, 2011; Sanjana *et al*., 2012).

Another *Xanthomonas* T3SE, XopD, is also localized to the nucleus and has DNA-binding activities (Fig. 1) (Kim *et al*., 2008). XopD has a modular architecture, with a helix–loop–helix (HLH) domain, followed by two EAR (ERF-associated amphiphilic repressor) motifs and a SUMO-protease domain (Kim *et al*., 2008). The presence of EAR motifs is particularly interesting as plant transcription factors with EAR motifs typically repress transcription of defence-related genes (Kim *et al*., 2008). Indeed, XopD represses defence-related genes *in vivo* despite the fact that XopD binds to DNA non-specifically *in vitro* (Kim *et al*., 2008). Additionally, XopD appears to target a transcription factor, MYB30, and represses its transcriptional activity (Canonne *et al*., 2011). However, XopD only requires the HLH domain in order to alter MYB30-regulated transcription, suggesting that XopD may use domains outside the HLH to target additional host proteins (Canonne *et al*., 2011). Future work characterizing the mechanisms by which XopD represses transcription will provide additional genomic tools to modulate eukaryotic transcription.

## Bacterial effectors that target RNA-binding proteins

HopU1 is a *P. syringae* T3SE that alters plant innate immunity. Like HopF2, HopU1 has ADP-RT activity. Therefore, to identify HopU1 targets, Fu and colleagues used a proteomics approach to identify plant proteins that are ADP-ribosylated by purified HopU1 (Fu *et al*., 2007). HopU1 targets a number of plant RNA-binding proteins (RBPs), including the glycine-rich RNA-binding protein GRP7 (Fig. 1) (Fu *et al*., 2007). These HopU1-targeted RBPs all share an RNA-recognition motif (RRM) necessary for RNA binding (Fu *et al*., 2007). HopU1 can ADP-ribosylate these RBPs both *in vivo* and *in vitro* (Fu *et al*., 2007). Characterization of how HopU1 modifies GRP7 provided mechanistic details on GRP7 function. Specifically, HopU1 ADP-ribosylates GRP7 at the arginine-49 (R49) residue within the RRM domain, which presumably disrupts GRP7-mediated RNA processing (Fu *et al*., 2007; Jeong *et al*., 2011; Woloshen *et al*., 2011). GRP7 appears to play a role in plant innate immunity, as the *grp7* mutant is more susceptible to *P. syringae* (Fu *et al*., 2007). This is potentially due to the decreased cell wall-based defences in the *grp7* mutant (Fu *et al*., 2007). Conversely, the overexpression of GRP7 increases resistance to *P. syringae* (Jeong *et al*., 2011). Furthermore, recent structural work has identified two unique loops in HopU1 that are essential for its enzymatic activities and GRP7-binding (Jeong *et al*., 2011). However, it is not clear which subset of defence genes is regulated by GRP7. Given that recent work has shown that plant defence genes are alternatively spliced during immune response, the identification of GRP7-regulated transcripts will provide further understanding of RNA processing in defence responses (Woloshen *et al*., 2011). Future characterization of GRP7 could also reveal a link between circadian rhythm and defence as GRP7 transcript and protein levels undergo circadian oscillations (Heintzen *et al*., 1997). Lastly, HopU1 may serve as a novel molecular tool to alter RNA transcript levels by targeting RNA-binding proteins.

## Conclusion

Characterizing the molecular mechanisms of effector functions has provided important insights into bacterial virulence and plant immunity. As we have seen with HopM1 and HopAR1, elucidating the effector function has led to the identification of novel components of plant cellular processes that contribute to plant immunity. In the case of HopM1, target identification has not only pulled out a previously uncharacterized ARF–GEF, AtMIN7, but also established a link between the secretory pathway and plant immunity. Similarly, HopAR1 target identification has shown the importance of the actin cytoskeleton in defence signalling. Furthermore, elucidating effector functions that target known signalling pathways has contributed to our existing knowledge of these pathways. For example, clarifying the molecular mechanisms by which HopF2 and HopAI1 modify their MAPK and MAPKK targets has identified key functional residues of these kinases. Lastly, while not presented in this review due to space constraints, the characterization of T3SE targets has also highlighted the crucial roles of proteins such as RIN4 and Pto in plant immunity (Martin *et al*., 1993; Tang *et al*., 1996; Bogdanove and Martin, 2000; Kim *et al*., 2002; 2005a,b; Ellis and Dodds, 2003; Mackey *et al*., 2003; Belkhadir *et al*., 2004; Liu *et al*., 2009; Oh and Martin, 2011).

T3SEs have enormous biotechnological potential to modulate eukaryotic transcription, rewire signalling pathways and influence various cellular processes. Future work on bacterial T3SEs will not only shed light on mechanisms of pathogenicity, but will also provide invaluable tools to effectively manipulate eukaryotic systems for pure and applied research. One of the major challenges of T3SE research will be to address their potential to have multiple targets and/or modify multiple pathways, which will require the identification and characterization of all potential T3SE targets in a eukaryotic cell. This information can then be used to rationally design efficient strategies to target specific processes and limit confounding off-target effects.
